# Supplementary material for: Perception of breast cancer risk factors: Dysregulation of TGF-β/miRNA axis in Pakistani females
Source: PLoS One. 2021 Jul 23;16(7):e0255243. doi: 10.1371/journal.pone.0255243 (PMC8301651; doi:10.1371/journal.pone.0255243)
Supplement: S1 Table — (PDF) [file pone.0255243.s001.pdf]

**S1 Table. Sequences of TFGβ pathway primers**

| Serial No. | Genes | Primer Sequences | GC Content | Product Size |
|---|---|---|---|---|
| **1** | TGFβ1-F | GGGACTATCCACCTGCAAGA | 55% | 239 bp |
| | TGFβ1-R | CCTCCTTGGCGTAGTAGTCG | 60% | |
| **2** | TGFβRII-F | AATGTGAAGGTGTGGAGACACTTA | 41.67% | 210 bp |
| | TGFβRII-R | AAGGCCAACTTCAAATCTTAACAC | 37.5% | |
| **3** | SMAD2-F | TTACAGACCCATCAAATTCAGAGA | 37.5% | 160 bp |
| | SMAD2-R | CTATCACTTAGGCACTCAGCAAAA | 41.67% | |
| **4** | SMAD4-F | GGTGCCTTTATGAGAATTGAGG | 45.45% | 181 bp |
| | SMAD4-R | TGTCTTCACCAAAGGTTTTCCT | 40.91% | |
| **5** | SMAD7-F | CTCGTTTGTGTTCTGTTTTGTTTC | 37.5% | 193 bp |
| | SMAD7-R | ACACACACTCCTGACAAGTGAAAT | 41.67% | |
| **6** | p21-F | ATGAAATTCACCCCCTTTCC | 45% | 164 bp |
| | p21-R | CCCTAGGCTGTGCTCACTTC | 60% | |
