# Supplementary material for: Perception of breast cancer risk factors: Dysregulation of TGF-β/miRNA axis in Pakistani females
Source: PLoS One. 2021 Jul 23;16(7):e0255243. doi: 10.1371/journal.pone.0255243 (PMC8301651; doi:10.1371/journal.pone.0255243)
Supplement: S2 Table — (PDF) [file pone.0255243.s002.pdf]

S2 Table. Sequences of miRNA Primers

Serial No.	miRNA	Primer Sequence
**1**	miR-29a-5p-F<br>miR-29a-5p-R	GCAGACTGATTTCTTTTGGTGT<br>GGTCCAGTTTTTTTTTTTTTTTCTGA
**2**	miR-140-F<br>miR-140-R	CAGCAGTGGTTTTACCCTATG<br>GGTCCAGTTTTTTTTTTTTTTTCTAC
**3**	miR-148a-F<br>miR-148a-R	CAGAAAGTTCTGAGACACTCC<br>CCAGTTTTTTTTTTTTTTTAGTCGGA
**4**	RNU6-F<br>RNU6-R	CTCGCTTCGGCAGCACA<br>AACGCTTCACGAATTTGCGT
