# Supplementary material for: Perception of breast cancer risk factors: Dysregulation of TGF-β/miRNA axis in Pakistani females
Source: PLoS One. 2021 Jul 23;16(7):e0255243. doi: 10.1371/journal.pone.0255243 (PMC8301651; doi:10.1371/journal.pone.0255243)
Supplement: S1 File — (PDF) [file pone.0255243.s003.pdf]

BREAST CANCER RESEARCH PROFORMA

1. Sample Type:

Collection Date ……………………

Hospital Name ……………………

☐ **Biopsy type**

- **Malignant tissue**
  - ☐ Fresh
  - ☐ FFPE
- **Normal /Benign tissue**
  - o Para-neuplastic junction / Tumor free margin area
  - o Epithelial / Connective & Stromal / Glandular tissue

☐ Pre-Chemo
☐ Post-Chemo

☐ **Blood Sample** Yes / No

- ☐ Whole Blood
- ☐ Serum/Plasma

2. Initial Data:

Patient Name ……………… **Age …………** **Contact No ……………**

Registration No……………….. **Bed No ………** **Ward No ……………...**

Locality……………………….. **Weight ………** **Blood group …………**

Date of Admission ……………. **Date of Operation ……………..**

Surgeon name ………………... **Pathologist name ………………**

Oncologist Name………………. **Date of Chemotherapy ……………….**

3. Screening Tests:

Did you ever perform screening tests and which kind?

☐ CBE ☐MMG ☐ USG ☐MRI ☐CT-Scan ☐Never
Date ……….. ………. …….. …….. ………..

How often do you go to breast cancer screening examinations?

CBE; ☐ After three month ☐ Six month ☐ Annually ☐Never

Mammography (MMG)

☐ Biannually ☐Annually ☐ Don't Performed

Would you please tell us why you have never had a breast cancer screening exam before?

☐ Fear or hesitation about examination ☐ Fear of finding some dangerous disease
☐ Doubts whether screening is effective ☐ Fear of radiation
☐ Fear of mastectomy ☐ Mammogram or other tests may be costly
☐ Fear of becoming burden on a family ☐No economical / moral support form family
☐ Lack of proper information ☐Poor diagnostic facilities in hospital.
☐ No time due to family issues ☐Lack of hospital paramedical and medical staff cooperation

4. Diagnostic Tests:

(A) Clinical Breast Exam (CBE) Yes / No

(B) Imaging Test Information Yes / No

Test Name	Yes	No	Lab. Name	ID
**MMG**				
**USG**				
**MRI**				
**CT-Scan**				
**PET**				

a) Hematological Test:

CBCs test Yes / No Clinical report

LFTs ALT ……. ALP ……… AST …………..

RFTs Yes / No Clinical report

ANC 1500 cells/mm3 1000 < 1500 500 - <1000/mm3 < 500/mm3

Cardiac history

- Insignificant
- Significant

Myocardial infarction	severe hypertension

Onset

Before Breast CA	After Breast CA
Insignificant relation with Breast CA	

Test

	Performed				No
Results	CK-MB	CK-BB	CK-MM	CPR	

Lipid Profile: Yes / No

Results	Triglyceride	Ref. value	Total cholesterol	R. value	HDL	R. value	LDL	R. value

b) Pathological Tests and Surgical Intervention:

i) Biopsy: **Dated**

Fine Needle Aspiration Cytology (FNAC) ……..

Needle Core Biopsy (NCB) ……..

Trucut Biopsy (TCB) ……..

ii) Open Surgery: Right Left both **Dated**

- Incision Biopsy ……...
- Excision Biopsy/Breast conserving surgery (BCS) or Lumpectomy ………
- Radical Mastectomy (Grade-I) ………
- Modified Radical Mastectomy (Grade II-III) ………
- Toilet or Partial Mastectomy (Stage-IV) ………

☐ Pre-Chemo Post-Chemo

Tumor Types:

- **Benign:**
  - Fibro-adenoma
- **Malignant**

DCIS IDC ILC

Invasive Ductal Carcinoma (IDC)

- **Tumor Grade**

Grade I Grade II Grade III

Tumor Markers (Hormone and Protein Receptor Test):

	+	-
☐ **ER**	☐	☐
☐ **PR**	☐	☐
☐ **HER2**	☐	☐

Hormonal Tests

a. Have you ever used female hormones (estrogen)? ☐ Yes ☐ No

b. Why do (did) you take estrogen? ☐ Menopausal symptoms ☐ Hysterectomy

☐ Bone problems ☐ cancers ☐ others (specify) ---------------------------

c. Age first took estrogen and how many year?------------------- ---------------

d. How did you take them? ☐ Injection ☐ Cream

**Hormones**	**Performed**			**Not performed**
	**Level in blood**	**Normal range**	**Date**	
**ER**				
**PR**				
**HER-2/neu**	**T**			

☐ **Tumor Staging:**

**0**	**I**	**II**	**III**			**IV**
			**III-A**	**III-B**	**III-C**	

iii) Stage based breast cancer therapy

- **Early, Localized or Operable (Stage I, Stage II, and operable Stage IIIA)**
  - Surgery
  - Postoperative Radiotherapy
    - External radiotherapy
    - Internal radiotherapy
      - Dose
      - Time duration
  - Pre / Postoperative systemic therapy

- **Late metastatic inoperable (Stage IIIB-C)**
  - **Chemotherapy**
    - Neo-adjuvent (before surgery)
    - Adjuvant (after surgery)
      - Drug type ………………………..
      - **Chemo-administration**

I.V	Oral

      - **Place**

Hospital	Home

      - **Coarse period** Cycle

04	06	08

      - Weeks **Time / Cycle**

04	06	08

      - **Time duration** Months

04	06	08

Breast-conserving surgery (BCS)

- Chemotherapy followed by surgery (BCS or total mastectomy), with lymph node dissection followed by radiation therapy.

- **Late metastatic inoperable (Stage IV)**
  - Surgery (MRM or TM), radiation therapy, or both
  - Hormonal $(ER/PR)^{+}$
    - Drug type…………………………. Dose/day …………………………
    - Time duration……………………..
    - Side effects ……………………….
  - Chemotherapy (ER/PR^{-})
    - Drug type ……………………….. Dose/day …………………………
    - Coarse ……………………….. Time duration …………………..
  - Monoclonal antibody / Molecular targeted therapy

Post treatment response / monitoring tests:

Post-surgery / chemo side effects

Recurrence after surgery / chemotherapy Yes No

**Years**	1-2Y 3-4Y 5Y > 5Y
**Survival after chemotherapy**	
**Years**	1Y 2Y 3Y >5Y

Post-surgery-molecular analysis ……………………………………………….

Post-chemo-molecular analysis ………………………………

Significant Risk Factors in Pakistani Population

1. Patient History

☐ **Initial notice of lump or tumor:**

☐ Change in size and shape of breast ☐ Hard or thickened inside

☐ Painful ☐ Painless ☐ Swell ☐ Warmth ☐ Irritation

☐ Sudden nipple discharge ☐ Redness or thickening of nipple or breast skin

☐ **Examination techniques**

☐ Breast self notice suddenly ☐ Self notice through regular breast exam

☐ Biannual or annual CBE ☐ Biannual or annual mammogram

☐ **Source of information about screening or diagnostic tests**

☐ Self aware ☐ Doctor ☐ Electronic / Social ☐ Media

☐ Relatives ☐ Family members ☐ Friends

☐ **First consultation**

☐ Husband if married ☐ Mother ☐ Elder female in family ☐ Medical doctor

☐ Homeopathic doctor ☐ Hakeem ☐ Spiritualist / Saint

☐ **Time elapse between initial notice and appropriate diagnosis and therapy**

☐ < 1 month ☐ 1-3 months ☐ > 6 months ☐ > 1 year

☐ **Age:**

- **At presentation** ☐
- **Menarche** ☐
- **Coitarche** ☐
- **Marriage** ☐

	Ist	2nd	3rd
**Pregnancy**	☐	☐	☐
**First live birth**	☐		
**Menopause**	☐		

2. Gynecological History Normal cycle Interrupted cycle

- **Menstrual cycle / month** | 2 3 > 3 |
- **Usual number of days of flow …………………**
- **If past menopause;**

Was your menopause ☐ Natural ☐ Artificial

☐ Painful ☐ Bleeding ☐ Painless and without bleeding

3. Personal History

Past surgical/breast CA	Past hist. of other disease	Co-infection	Neo-adjuvant / adjuvant therapy

4. Personal History of other Diseases

		Before Breast CA	After Breast CA
Typhoid and Paratyphoid fever	Hepatitis B or C	………………..	……………….
Diabetes	Cerebrospinal Meningitis	………………..	……………….
Blood Pressure	Hypertension	………………..	……………….
Tuberculosis	Rectal / colon polyp	………………..	……………….
Gall / kidney / bladder stones	Liver Cirrhosis	……………….	……………….
Gynecological problems	Breast cyst	……………….	……………….
Chronic Bronchitis	Stomach ulcer	……………….	……………….

5. Family History

**Breast cancer**					**Other cancer**				
**First degree relative**				**2nd deg./ other**	**First degree relative**				**2nd deg./other**
Parents	Spouse	Sibling	Brother /Sister		Parents	Spouse	Sibling	Brother /Sister	
**Age**					**Age**				
**Number of affected relatives**					**Number of affected relatives**				

6. Marital Status

- Single
- Married
  - Separated / Divorced
  - Widowed

Patient's marriage relation

Within family	Out of family

- **Patients family marriage relation**

Ist cousin	2nd cousin	Other relation

- **Parents marriage relation**

Within family	Out of family

- **Parents family marriage relation**

Ist cousin	2nd cousin	Other relation

- **Pregnancy status**

Normal delivery	With operation	
	Minor	Major

7. No. still birth (carried 5 months or more)? Yes No

10. No. of miscarriage (carried less than 5 months)? Yes No

a. Use DES (Diethylstilbestrol) or nay other to prevent miscarriage Yes No

b. Age at usage ………. Dosage …………….

c. No. of month's usage ……….

8. No. of Children

Nulli-parity	Multi-parity	
0	1 – 3	> 3

9. Lactation Period

Ist	2nd	3rd	Average

10. Life Style

- **Environment**

Rural	Peri-urban	Urban

- **In your work place of daily life are (were) you regularly exposed to any of the following**

☐ Chemical/acid/solvents ☐ Coal or stone dust ☐ Diesel engine exhaust

☐ Gasoline exhaust ☐ Factory smoking ☐ Textile fiber / dust

☐ Wood dust ☐ Herbicides / pesticides ☐ X-rays/ radioactive materials

- **Diet**

Frequent veg. eater		Frequent meat eater		Veg + meat + fruit eater
Green veg.	Non green	White meat	Red meat	

- **Current physical condition**

Type of exercise patient get on each day

☐ Yoga ☐ Gym at home ☐ Gym at gymnasium ☐ None

☐ Limbs movements on daily basis ☐ Just kitchen activity

Time duration of exercise per day

☐ 15-30 minutes ☐ 30-45 minutes ☐ > I hours

- **Education**

	Patient	Husband	Family
**i)** Illiterate	☐	☐	☐
**ii)** Literate	☐	☐	☐
Primary	☐	☐	☐
Middle	☐	☐	☐
Matric	☐	☐	☐
Intermediate or above	☐	☐	☐

- **Socio-economic status**
  - **Average monthly income** (from all sources)

Poor class (10,000-25,000)	Average class (25,000-50,000)	Upper class (>50,000)

- **Occupation of Patient**

<table>
<tr><td colspan="2">House wife</td><td>Laborer</td><td colspan="2">Jobs</td></tr>
<tr><td rowspan="3">Working</td><td rowspan="3">Non-working</td><td rowspan="3"></td><td colspan="2">Job Sectors</td></tr>
<tr><td>Government</td><td>Private</td></tr>
<tr><td colspan="2">Institution name</td></tr>
<tr><td colspan="3">Other comments if any:</td><td></td><td></td></tr>
<tr><td colspan="3" rowspan="6"></td><td colspan="2">Designation / Grade</td></tr>
<tr><td></td><td></td></tr>
<tr><td colspan="2">Nature of Job</td></tr>
<tr><td></td><td></td></tr>
<tr><td colspan="2">Total Job hours</td></tr>
<tr><td></td><td></td></tr>
</table>

☐ **Occupation of Husband** (if married)

<table>
<tr><td>Unemployed</td><td>Laborer</td><td>Business</td><td colspan="2">Jobs</td></tr>
<tr><td></td><td></td><td></td><td colspan="2">Job Sectors</td></tr>
<tr><td colspan="3" rowspan="10">Other comments if any</td><td>Government</td><td>Private</td></tr>
<tr><td colspan="2">Institution name</td></tr>
<tr><td></td><td></td></tr>
<tr><td colspan="2">Designation / Grade</td></tr>
<tr><td></td><td></td></tr>
<tr><td colspan="2">Nature of job</td></tr>
<tr><td></td><td></td></tr>
<tr><td colspan="2">Total Job hours</td></tr>
<tr><td></td><td></td></tr>
</table>

☐ **Occupation of Parents** (if un-married)

**Unemployed**	**Laborer**	**Business**	**Jobs**	
			Job Sectors	
Other comments if any:			Government	Private
			Institution name	
			Designation / Grade	
			Nature of Job	
			Total Job hours	

11. Stress Factors

**Normal**	**Acute / Short time Stress**	**Chronic / Long time Stress**
**1) External stress source**<br>i) Yes N<br>ii) Yes No<br>iii) Yes No<br>iv) Yes No<br>v) Yes No<br><br>**A) Work related stress**<br>i) Yes No<br>ii) Yes No<br><br>**B) Life event stress**<br>i) Yes No<br>ii) Yes No<br>iii) Yes No<br>iv) Yes No<br><br>**C) Every day or total stress**<br>i) Yes No<br><br>**2) Internal stress source**<br>i) Yes No	**Physiological Stress**<br>i) Headaches<br>ii) Stomach aches or indigestion<br>iii) Heart palpitations<br>iv) High blood pressure<br>v) Shortness of breath<br>**Psychological Stress**<br>i) Apprehension<br>ii) Fear of weight and hair loss	**1) External stress source**<br>i) Stress of poverty or financial stress<br>ii) Stress of dysfunction of family or ongoing illness in the family.<br>iii) Socially isolated or grouped house female<br>iv) Fear of disease treatment<br>v) Fear of disease progression or recurrence<br><br>**A) Work related stress**<br>i) Unemployment<br>ii) Serious problems at work or job place being / faced herself or his husband<br><br>**B) Life event stress**<br>i) Stress of being trapped in unhappy marriage or in a despised of jobs/career<br>ii) Traumatic event in early childhood<br>iii) Recent loss of spouse, parents, child or loved one or divorced<br>iv) Involvement in a serious accident<br>**C) Every day or total stress**<br>i) Poorly managed/ignored everyday stressors<br><br>**2) Internal stress source**<br>i) Long history of anxiety or depression

Psychological outcomes

Healthy Subjects

- Disease onset Yes No

Subjects with Disease

Progression / severity — Recovery hindrance / Drug resistance

Recurrence / relapses

Post-psychological chronic stressor patients

Level of Androgen ……………… — Level of ER/PR ………………….

NK cell activity …………………. — Level of cortisol …………………

Immunity Status ………………… — Tumor grade …………………….

Psychological problems		Date	Treated / untreated
Manic Psychosis	Paranoid Psychosis	………………..	……………….
Hallucinatory	Schizophrenia	………………..	……………….

Trivial Factors in Pakistani Population

**Alcoholic**	Yes / No
**Smoking**	Yes / No
**Hormone Replacement Therapy (HRT)**	Yes / No
**Hysterectomy**	Yes / No
**Oopherectomy**	Yes / No
**Birth control methods**	Yes / No

If yes indicate at age when first used and number of year used?

Method used	Age	Years
Rhythm	……	…….
Diaphragm	……	…….
Cream / foam / jelly	……	…….
Intra-uterine device (IUD)	……	…….
Condom (partner)	……	…….
Vasectomy (partner)	……	…….
None		

(M.Phil Scholar)

(PhD Scholar)

Head Surgery / Pathology
(Hospital)

Dr. Bushra Ijaz
Applied and Functional Genomics (CEMB)
