# Supplementary material for: Perception of breast cancer risk factors: Dysregulation of TGF-β/miRNA axis in Pakistani females
Source: PLoS One. 2021 Jul 23;16(7):e0255243. doi: 10.1371/journal.pone.0255243 (PMC8301651; doi:10.1371/journal.pone.0255243)
Supplement: S2 File — (PDF) [file pone.0255243.s004.pdf]

چھا تی کے سرطان کا تحقیقی فارم

.1 نمونے کی قسم :---------------- اکٹھا کرنے کی تاریخ:-----------

Hospital Name ……………………

جید کی قسم ☐
• مہلک بافتہ
کیمو تھیراپی سے پہلے ☐
کیمو تھیراپی کے بعد ☐
تازہ ☐
فارمالین میں محفوظ ☐
• عام / بے زرر بافتہ
خون کا نمونہ ☐ Yes / No
سیال /سیرم ☐ مکمل خون ☐

2 :ابتدا یہی مواد

مریض کا نام ………………… عمر ………… فون نمبر ……………
رجسٹریشن نمر……………….... بستر نمبر ……… وارڈ نمبر ……………...
رہا یش……………………….. وزن ……… خو ن کا گروپ …………
داخلے کی تاریخ ……………. جرا ہی کی تاریخ ……………..
جراح کا نام …………………... ماہر تشخیص……………….
ماہر سرطان………………… کیمو تھیراپی کی تاریخ………………

3 :اسکرییننگ ٹیسٹ

کیا آپ نے کبھی اسکرییننگ ٹیسٹ کرواۓ اور کس قسم کے ؟

☐ CBE ☐ MMG ☐ USG ☐ MRI ☐ CT-Scan ☐ Never
تاریخ ……….. ………. …….. …….. ………..

آپ کتنی بار چھا تی کے معا نے کے لیے جاتے ہیں؟

CBE ☐ ۳ مہینے بعد ☐ 6 مہینے بعد ☐ سالا نہ کبھی نہیں ☐
Mammography (MMG) ☐ 6 مہینے بعد ☐ سالا نہ کبھی نہیں ☐ ☐

کیا آپ بتا سکتی ہیں کہ آپ نے چھا تی کا معا نہ کیوں نہیں کروا یا ؟
مہلک بیما ری کا خوف
معا ینے کے بارے میں خو ف اور ہچکچاہٹ
معا ینے کے بارے میں خدشا ت کہ اسکا کو ہی فا یدہ نہیں

آپریشن کا خوف
میمو گرام اور دوسرے ٹیسٹوں کی لاگت کا ڈر
خا ندان پہ بو جھ بننے کا خوف
خا ندان کی معا شی اور اخلا قی ہمایت کا نہ ہو نا
مکمل معلو مات کا فقدان
ہسپتال میں مکمل تشخیصی سہو لیا ت کا فقدان
خا ندانی مسایل کی وجہ سے وقت کا نا ملنا

4. تشخیصی ٹیسٹ .

(A) چھاتی کا معا ئنہ ہا ں/نہیں

(B) تصویروں کی پرکھ کے بارے میں معلومات ہا ں/نہیں

ٹیسٹ کا نا م	ہا ں	نہیں	لیبا رٹری	شنا خت
MMG				
USG				
MRI				
CT-Scan				
PET				

a) :خو ن کے ٹیسٹ

طبی رپورٹ ہا ں/نہیں خو ن کےخلیو ں کے ٹیسٹ

جگر کے افعال ALT ……. ALP ……… AST …………..
گردے کے افعال ہا ں/نہیں طبی رپورٹ
ANC > 1500 cells/mm3 >1000 < 1500 500 - <1000/mm3 < 500/mm3
دل کے امرض کا پس منظر
غیر اہم ☐
اہم ☐
شدید ہا ئی بلڈ پریشر دل کے نسیجو ں کا بیکا ر ہو نا

واقع ہوا

چھا تی کے سرطان کے بعد چھا تی کے سرطان سے پہلے

چھا تی کے سرطان سے تعلق نہیں ہے

ٹیسٹ

نہیں	کیا جا چکا ہے				
	CPR	CK-MM	CK-BB	CK-MB	نتا یج

ہا ں/نہیں :کو لیسٹرول کے ٹیسٹ

نتا یج	Triglyceride	Ref. value	Total cholesterol	R. value	HDL	R. value	LDL	R. value

a) تشخیصی ٹیسٹ اور جراہی مداخلت:

i) جید: تاریخ

□ Fine Needle Aspiration Cytology (FNAC) ……..

□ Needle Core Biopsy (NCB) ……..

□ Trucut Biopsy (TCB) ……..

ii) جسمانی جراحت: سیدھی الٹی دونوں اطراف سے تاریخ

چیرہ تشخیص ……...

قطع برید تشخیص/ چھا تی کی محفو ظ جراہت ………

پستان براری ………

جد ید پستان براری ………

جزوی پستان براری ………

رسولی کی اقسام: کیمو تھیراپی سے پہلے / کیمو تھیراپی کے بعد

بے ضرر: □

ریشہ دار و غدود دار

مہلک □

DCIS	IDC	ILC

Invasive Ductal Carcinoma (IDC)

رسولی کا درجہ □

درجہ I	درجہ II	درجہ III

رسولی کے مارکر : ہارمون اور لحمیہ وصول کے جانچ پڑتال

	+	-
□ ER		
□ PR		
□ HER2		

ہا رمو ن کی جانچ پڑتال

a. کیا آپ نے کبھی خواتین کے ہا رمو ن استعما ل کیے ہیں ؟ ہا ں نہیں

b. آپ نے ایسٹروجن کیوں کھا یی رجو نورتی علامات رحم کے اپریشن کے بعد

c. کو ئی اور وجو ہات ________________ سرطان ☐ ہڈیوں کے مسا ئل کے دود

کس عمر میں ایسٹروجن کا استعمال کر نا شروع کیا---

d. ایسٹروجن کس شکل میں استعمال کی ؟

کریم ٹیکہ

ہارمون	انجا م کردہ			نہ انجا م کردہ
	خون میں سطح	ٹھیک حد	تا ریخ	
ER				
PR				
HER-2/neu ☐	T			

رسولی کا مرحلہ ☐

0	I	II	III			IV
			III-A	III-B	III-C	

iii) مرحلہ کی بنیا د پر چھا تی کے سرطان کا علا ج

جلد / مقامی / قابل جراہی مرحلہ ☐

جراہ ☐

بعد از اپریشن شعاعی علا ج ☐

بیرونی شعاعی علا ج ☐

اندرونی شعاعی علا ج

خوراک

دورانیہ وقت ☐

قبل از / بعد از اپریشن منظم علا ج ☐

طویل انتقال مرض نا قابل برداشت ☐

کیمیا ہی علا ج ☐

عمل جراحت سے پہلے ☐

عمل جراحت کے بعد ☐

دوائی کی قسم ________________

دوائی کا انتظا م

I.V	Oral

Hospital	Home

Cycle ________________

04	06	08

جگہ

مدت / جگہ

چکر

وقت

04	06	08

مہینے

04	06	08

چھا تی کی محفو ظ جراہت ☐

کیمیا ئی علا ج جراحت کے بعد ، لیمف نوڈ کو چاک کرنے کے بعد شعا عی علا ☐

نا قابل جراحت دیر ہو چکا پھیلا ہوا سرطان کا مرحلہ [درجہ چہا رم] ☐

یا دونوں ,شعاعی علاج ,(MRM or TM) عمل جراحت ☐

Hormonal (ER/PR) + ☐

o دوائی کی قسم............................... دن /خوراک ....

o دورانیہ وقت.......................... o منفی اثرات ....

کیمیا ئی علا ج (ER/PR-) ☐

o دوائی کی قسم .... دن /خوراک..............................

o دورانیہ وق............................ منفی اثرات ....

☐

مالیکیو لر ٹارگٹ علا ج / یک قلمیہ دافع جسم ☐

نگرانی کے ٹیسٹ / بعد از علا ج جسما نی رد عمل: ☐

بعد از جراحت / کیمیا ئی علا ج منفی اثرات

ہا ں / نہیں بعد از جراحت دوبارہ واقع ہوا

سال

1-2Y	3-4Y	5Y	> 5Y
	Yes		No

کیمیا ئی علاج کے بعد بقا

سال

1Y	2Y	3Y	>5Y

جراحت کے بعد سالماتی تجزیہ ....

یمیا ئی علا ج کے بعد کا سالماتی تجزیہ ....

پا کستا نی آبادی کو درپیش خطرے کے اہم عوامل

1. مریض کی طبی تاریخ

رسو لی اور گانٹھ کا ابتدائی نو ٹس: ☐

اندر سے سخت اور مو ٹا   چھاتی کے جسامت اور شکل میں تبد یلی

جلن   حرارت   سوجن   بے درد   تکلیف دہ

ماده کا نپل سے اچانک خا رج ہو نا

چھا تی کی جلد میں یا نپل میں سرخی اور سوجن

معا ئینہ کرنے کا طریقہ کا ر

چھا تی کا اچانک سے از خود محسوس کرنا

سا لا نہ یا دو سالہ سی بی ای

سا لا نہ یا دو سالہ میمو گرام

با قا عدہ چھا تی کے معائینہ میں پتا لگنا

اسکرینینگ یا تشخیصی ٹیسٹ کی معلو مات کے ذرائع ☐

میڈیا ☐   الیکٹرانک یا سماجی ☐   ڈاکٹر ☐   خود آگا ہی

دوست ☐   گھر کے افراد ☐   رشتہ دار

پہلی بار مشورہ ☐

صوفی / روحا نی بابا ☐   حکیم ☐   ہو میو پیتھک معالج ☐   طبی معالج ☐   گھر میں بڑی خاتون ☐   والدہ ☐   خا وند

منا سب تشخیص اور علا ج کا درمیا نی وقفہ   مہینا 1-3 ☐   مہینا 1 > ☐

عمر:

ظا ہر ہونے پر ☐   > 1 year   > 6 months ☐

ماہواری ☐

پاکیزگی ☐

شادی ☐

	١st	٢	٣r
حمل			

پہلی زندہ اولاد ☐

رجو نورتی ☐

2. دایہ گیری کی تاریخ

ماہ واری کے تسلسل میں رکاوٹ

ماہ واری کا تسلسل / ☐   2   3   > 3

بہاؤ کے دنو ں کی تعداد .... ☐

اگر رجو نورتی گزر چکا ہے; ☐

مصنو ئی ☐   قدرتی ☐   رجو نورتی

بے درد اور بغیر خون بہے ☐   خون بہنا ☐   تکلیف دہ ☐

3. مریض کی زاتی ہسٹری

جراہت کے بعد چھا تی کا سرطان	دوسری بیماریو ں کی تفصیل	شریک انفیکشن	نو ضمیر/ ضمنی علاج

4. دوسری بیماریو ں کی تفصیل

دوسری بیماریو ں کی تفصیل		چھا تی کے سرطان سے پہلے	چھا تی کا سرطان کے بعد
ٹائیفا یڈ بخار	ہیپا ٹائٹس بی یا سی	………………..	……………….
ذیابیطس	دماغی اسپائنل مینیجا ئٹس	………………..	……………….
فشار خون	ہا ئپر ٹینشن	………………..	……………….
ٹی بی	بڑی آنت میں پٹی	………………..	……………….
مثا نے/گردے میں پتھری	جگر کا سروسس	……………….	……………….
دایہ گیری کے مسائل	چھاتی میں گلٹی	……………….	……………….
جان لیوا پیپھڑوں کی بیماری	معدے کا السر	……………….	……………….

5. خاندان کی تفصیل

<table>
<tr><td colspan="5">چھا تی کا سرطان</td><td colspan="5">دوسرا سرطا ن</td></tr>
<tr><td colspan="4">بہت قریبی رشتہ دار</td><td>دور کے رشتہ دار</td><td colspan="4">بہت قریبی رشتہ دار</td><td>دور کے رشتہ دار</td></tr>
<tr><td>واالدین</td><td>شریک حیا ت</td><td>بہن بھا ئی</td><td>بھا ئی بہن/</td><td></td><td>واالدین</td><td>شریک حیا ت</td><td>بہن بھا ئی</td><td>بھا ئی بہن/</td><td></td></tr>
<tr><td colspan="5">عمر</td><td colspan="5">عمر</td></tr>
<tr><td></td><td></td><td></td><td></td><td></td><td></td><td></td><td></td><td></td><td></td></tr>
<tr><td colspan="5">متا صرہ رشتہ دارکی تعداد</td><td colspan="5">متا صرہ رشتہ دارکی تعداد</td></tr>
<tr><td></td><td></td><td></td><td></td><td></td><td></td><td></td><td></td><td></td><td></td></tr>
</table>

6. ازدواجی حیثیت

کنواری ☐
شا دی شدہ ☐
o علیہدگی / طلاق یا فتہ
o بیوہ

مریض کا ازدواجی رشتہ

خاندان سے باہر	خاندان کے اندر

o **مریض کے خاندان کا ازدواجی تعلق**

کو ئی اور رشتہ	دوسرہ کزن	پہلا کزن

o **والدین کی شادی ہوئی**

خاندان سے باہر	خاندان کے اندر

o **والدین کی شادی ہوئی**

کو ئی اور رشتہ	دوسرہ کزن	پہلا کزن

o **حمل کے بعد**

اپریشن کے ساتھ		وضع حمل
بڑا	چھوٹا	

7. مردہ بچہ ہاں / نہیں
:۹ اسقا ط حمل کی تعداد ہاں / نہیں
a. اسقا ط حمل کو روکنے کے لیے ڈی ای ایس استعمال کیا ہاں / نہیں
b. استعمال کے وقت عمر .... خوراک ....
c. جتنے مہینے استعمال کیا ....

8. **بچوں کی تعداد**

-	کثیر زئی	بے اولا د
> 3	1 – 3	0

9. **بچے کو چھا تی سے دودھ پلایا**

Average	3rd	2nd	Ist

10. **زندگی کا طرز عمل**

ماحول ☐

شہری	قصباتی	دیہا تی

اپنی روز مرہ زندگی میں کیا آپکا سامنا ان چیزوں سے ہوا ؟ ☐

تیزاب

کو ئلہ یا پتھر وں کی دھول

پٹرول کا دھواں

کا رخانے کا دھواں

کپڑے کا رووا ں

اکس رے / تابکاری مادے

کیڑے مار ادویات

خوراک ☐

سبزی + گوشت + پھل	زیا دہ گوشت کھاتے ہیں		زیادہ سبزیاں کھاتے ہیں	
	سرخ گوشت	سفید گوشت	دوسری سبزیاں	ہرے پتوں والی سبزی

مو جو دہ جسمانی صحت ☐

روزمرہ ورزش

کو ئی نہیں　جم میں ورزش　گھر میں ورز ش　یوگا

رومرہ ہا تھ پاوں کی　حرکا ت　باورچی خا نے کا کا م

روزمرہ ورزش کا دورانیہ

گھنٹہ < I　☐مینٹ 30-45　☐ مینٹ 15-30　☐

تعلیم ☐

خاندان　شوہر　مریض

		ان پڑھ (i
		پڑھا لکھا \ (ii
		پرائمری (iii
		مڈل (iv
		میٹرک (v
		انٹر یا اس سے زیادہ (vi

معا شرتی و معاشی حالات ☐

اوسط ماہانہ آمدن ☐

امیر طبقہ (>50,000)	درمیا نہ طبقہ (25,000-50,000)	غریب طبقہ (10,000-25,000)

مریض کا پیشہ ☐

نوکری	مزدور	گھریلو خاتتون	
پیشہ		کام نہیں کرتی	

نجی	سرکاری			کام کرنے والی
ادارے کا نام				
		تبصرہ		
گریڈ				
نوکری کی نو عیت				
نو کری کے کل گھنٹے				

شوہر کا پیشہ □

نوکری		کاروبار	مزدور	بے روزگار
پیشہ				
نجی	سرکاری	تبصرہ		
ادارے کا نام				
گریڈ				
نو کری کے کل گھنٹے				
نوکری کی نو عیت				

والدین کا پیشہ □

نوکری		کاروبار	مزدور	بے روزگار
پیشہ				
نجی	سرکاری	تبصرہ:		
ادارے کا نام				
گریڈ				
نو کری کے کل گھنٹے				
نوکری کی نو عیت				

11. ذہنی دباؤ کے عوامل

دائمی یا لمبے عرصے کا دباؤ	شدید / تھوڑے وقت کا دباؤ	عام
بیرونی عوامل (1<br>۱. غربت یا معاشی دباؤ<br>۲. خاندان میں موجد میباریوں کا دباوۃ<br>۳. معاشرتی طور پر علیہدہ یا مخلوط خاندان میں رہنے کا دباؤ<br>۴. علاج کا خوف<br>۵. بیماری دوبارہ ہونے کا خوف یا اس میں بڑہا ؤ کا خوف<br><br>۲. کام سے متعلق زہنی دباؤ<br><br>۱ بے روزگاری<br>۲. کام می جگہ پر شدید مسا ئل<br><br>۳. زندگی کی پریشانیا ں<br>۱. مرضی کے شادی یا نوکری میں پھنسنے کا خوف<br>۲. بچپن کا کو ئی تکلیف دہ واقع<br>۳. قریبی رشتہ داروں کے ساتھ ہادثہ<br><br>۴. پریشانی اور ذہنی دباؤ کی ہسٹری	**جسمانی تکا لیف**<br><br>سردرد<br>معدہ ا درد<br>نظام انہظام<br>دل کی لرزش<br>فشار خون<br>سانس کی رکاوٹ<br><br>**دماغی دباؤ**<br><br>خدشات<br>وزن اور بال گرنے خوف	**بیرونی عوامل (1**<br>ہا ں / نہیں (i<br>ہا ں / نہیں (ii<br>ہا ں / نہیں (iii<br>ہا ں / نہیں (iv<br>ہا ں / نہیں (v<br>**کام کے متعلق دباؤ (A**<br>ہا ں / نہیں (i<br>ہا ں / نہیں (ii<br>**زندگی کے واقعات کا دباؤ (B**<br>ہا ں / نہیں (i<br>ہا ں / نہیں (ii<br>ہا ں / نہیں (iii<br>ہا ں / نہیں (iv

نفسیا تی نتائج

صحت مند لوگ

نہیں   ہاں   بیما ری کا آغاز

بیما ر لوگ

بحا لی میں رکاوٹ   شدت

دوبارہ ظہور

بعد از ندسیا تی دایمی ذہنی دبا ؤ کے مریض

ER/PR کی سطح ....   انڈروجن کی سطح ....

کورٹیسول کی سطح....................   NK خلیہ کا کام ....

رسولی کا درجہ........................   قوت مدافعت....................

نفسیا تی مسائل

شقاق دماغی   پاگل دماغی حالت   سحر انگیزی

پاکستانی آبادی میں معمولی عوامل

شراب نو شی	ہاں/ نہیں
سگرٹ نوشی	ہاں/ نہیں
ہارمون رپلیسمنٹ تھیراپی	ہاں/ نہیں
رحم کا اپریشن	ہاں/ نہیں
بیضہ دانی کا اپریشن	ہاں/ نہیں
پیدائش پر قابو پانے کے طریقے	ہاں/ نہیں

کس عمر میں اور کتنا عرصہ استعمال کیا

طریقہ استعمال	عمر	سال
	……	…….
پردہ شکم	……	…….
کریم	……	…….
(IUD)	……	…….
کونڈم	……	…….
وسیکٹومی	……	…….

کوئی نہیں

(پ-ایچ-ڈی سکالر) (ایم فل سکالر)

جراہی شعبے کے سربراہ

اطلاقی باضابطہ مو رثیت لیبا رٹری (ہسپتال)
