# Supplementary material for: Perception of breast cancer risk factors: Dysregulation of TGF-β/miRNA axis in Pakistani females
Source: PLoS One. 2021 Jul 23;16(7):e0255243. doi: 10.1371/journal.pone.0255243 (PMC8301651; doi:10.1371/journal.pone.0255243)
Supplement: Attachment — Submitted filename: Comments_SM_PLOS.pdf [file pone.0255243.s005.pdf]

Comments:

In the manuscript ‘Perception of breast cancer risk factors: Dysregulation of TGF-β/miRNA axis in Pakistani females’, Fayyaz etal., highlighted the different risk factors like stress, no. of children, menstrual cycles and correlated with age, stage and grade etc. of the diseases. The important thing is they performed in silico and study to pick the miRNA regulating the TGF beta pathway and confirmed the expression in clinical samples both in blood as well as biopsy. The results can help in designing the therapeutic strategy and monitoring the disease state in the population for better output. The manuscript is acceptable for publication after incorporating following revisions.

1. In abstract authors stated that miRNA ‘significantly dysregulated’, it is better to mention either the P value or the fold change observed in the study.
2. In introduction highlight and compare the risk factors of breast cancer observed in current study with that of other Asian country data.
3. In materials and methods section primer sequences used for expression analysis of TGF-beta pathway genes and miRNA are not provided. Supply it as supplementary file.
4. Result should be concluded with ‘May or can’ as more studies are required on large data set to be sure to state.
5. Under heading ‘miRNA-target genes network analysis’, where authors mentioned the ‘different cascades’, it’s better to name few important one.
6. Improve the quality of figure 6.
7. Carefully remove the typo-errors from the manuscript.

Tenured Assoc. Prof. Sobia Manzoor, PhD
Head of the Molecular Virology research Group
Department of Healthcare Biotechnology
Atta-ur-Rahman School of Applied Bio-Sciences (ASAB)
National University of Sciences and Technology (NUST)
H-12, Islamabad 44000, Pakistan; Office: +92 51 90856147
Email: lcianunique@yahoo.com; dr.sobiamanzoor@asab.nust.edu.pk
http://www.nust.edu.pk/INSTITUTIONS/Schools/ASAB/Departments
https://www.ncbi.nlm.nih.gov/pubmed/?term=sobia+manzoor+et+al
scholar.google.com/citations?view_op=list_works&hl=en
