# Supplementary material for: Perception of breast cancer risk factors: Dysregulation of TGF-β/miRNA axis in Pakistani females
Source: PLoS One. 2021 Jul 23;16(7):e0255243. doi: 10.1371/journal.pone.0255243 (PMC8301651; doi:10.1371/journal.pone.0255243)
Supplement: Attachment — Submitted filename: Response to reviwers.docx [file pone.0255243.s006.docx]

| **Editors Comments** | | | |
| --- | --- | --- | --- |
| **Sr. No** | **Comment** | **Response** | **Page Number** |
| 1. | Please ensure that your manuscript meets PLOS ONE's style requirements, including those for file naming. | Revised manuscript is set according to the PLOS ONE’s style. |  |
| 2. | Please review your reference list to ensure that it is complete and correct. If you have cited papers that have been retracted, please include the rationale for doing so in the manuscript text, or remove these references and replace them with relevant current references. Any changes to the reference list should be mentioned in the rebuttal letter that accompanies your revised manuscript. If you need to cite a retracted article, indicate the article’s retracted status in the References list and also include a citation and full reference for the retraction notice. | The reference list is thoroughly checked There is no retracted article cited in the manuscript. |  |
| 3. | Please include additional information regarding the survey or questionnaire used in the study and ensure that you have provided sufficient details that others could replicate the analyses. For instance, if you developed a questionnaire as part of this study and it is not under a copyright more restrictive than CC-BY, please include a copy, in both the original language and English, as Supporting Information | The questionnaire used in the study is provided as supplementary file S1 (English) and S2 (in original Language. | Please see the S1 and S2 files |
|  |  |  |  |

| **REVIEWER 1** | | | |
| --- | --- | --- | --- |
|  | **Comment** | **Response** | **Page No.** |
| 1. | In abstract authors stated that miRNA ‘significantly dysregulated’, it is better to mention either the P value or the fold change observed in the study | Thank you, reviewer, for the comment. The P value has been added to indicate significant change in the abstract.  Please see the revised manuscript abstract, addition is highlighted in green | Please see page No. 2. Line # 41. |
| 2. | In introduction highlight and compare the risk factors of breast cancer observed in current study with that of other Asian country data. | The comparison is added in the revised manuscript introduction highlighted in green. | Please see page No. 3, line # 64-67,  Ref 7-10. |
| 3. | In materials and methods section primer sequences used for expression analysis of TGF-beta pathway genes and miRNA are not provided. Supply it as supplementary file. | The primer sequences have been provided as supplementary file S1 and S2 Tables in revised manuscript | Please see the S1 and S2 Tables |
| 4. | Result should be concluded with ‘May or can’ as more studies are required on large data set to be sure to state. | The results have been concluded with either may or can.  Please see the revised manuscript the changes have been highlighted in green | Page No. 2, 22, and 23, 25-27. Line # 45, 370, 380, 383, 429, 484 and 487. |
| 5. | Under heading ‘miRNA-target genes network analysis’, where authors mentioned the ‘different cascades’, it’s better to name few important one. | The miRNA target cascades have been added under the heading ‘miRNA-target genes network analysis’. Please see the revised manuscript the changes have been highlighted in green. | Page NO.17, line # 255-257. |
| 6. | Improve the quality of figure 6. | The quality of figure 6 has been improved in revised manuscript. | Please see figure 6. |
| 7. | Carefully remove the typo-errors from the manuscript. | The revised manuscript has been thoroughly checked and edit to remove the typo-errors |  |

| **REVIEWER 2** | | | |
| --- | --- | --- | --- |
|  | **Comment** | **Response** | **Page No.** |
| 1. | The reaction conditions for the synthesis of miRNA cDNA should be given. | The reaction condition has been added in the revised manuscript. Please see the heading synthesis of miRNA cDNA. The addition is highlighted in yellow | Page No. 9, Line # 174-177. |
| 2. | Where possible provide references for the methods used in the study. | References has been added in the revised manuscript materials and methods highlighted in yellow. | Please see page No. 8 line # 163 and Page # 9, line No. 184. |
| 3. | In Table 2 authors do not need to mention Pearson correlation with each parameter studied as this information is already provided in title of the Table 2. | Table 2 has been revised as suggested by the reviewer. The changes have been highlighted in yellow. | Please see Table 2 at page No.15 & 16. |
| 4. | In Table 2 mention r and p-value with respective values of the correlation study. | Table 2 has been revised as suggested by the reviewer. The changes have been highlighted in yellow. | Please see Table 2 at page No.15 & 16. |
| 5. | In result section under heading ‘Demographic Profiling of Breast Cancer Patients’ remove the word elucidation with observed. | The word elucidation is replaced with observed. This change has been highlighted in yellow in the revised manuscript | Please see page No. 14. Line #238 |
| 6. | In discussion section compare your results with other Asian countries data to highlight the differences if any observed. | The information is added in the revised manuscript. The addition is highlighted in yellow in revised manuscript. | Please see page No. 23. Line # 388, 397-399. And Ref No. 40, 44 and 45. |
| 7. | It is suggested to mention the number of experimental repeats in figure legends. | The number of experimental repeats has been added in the revised manuscript, highlighted in yellow. | Please see figure 4 legend at page No. 20, line # 320 and Figure 5 legend at page No. 21 line # 337. |
| 8. | Improve the quality of figures. | Quality of Figures have been improved in the revised manuscript. |  |
| 9. | Remove the typo errors from the manuscript. | The typo-errors have been removed from the revised manuscript. |  |
